# Skin AGEs and diabetic neuropathy

**DOI:** 10.1186/s12902-021-00697-7

**Published:** 2021-02-23

**Authors:** Stella Papachristou, Kalliopi Pafili, Nikolaos Papanas

**Affiliations:** Diabetes Centre-Diabetic Foot Clinic, Second Department of Internal Medicine, Democritus University of Thrace, University Hospital of Alexandroupolis, G. Kondyli 22c, 68100 Alexandroupolis, Greece

**Keywords:** Advanced glycation end-products, Autofluorescence, Complications, Diabetes mellitus, Microvascular, Neuropathy

## Abstract

Advanced glycation end-products (AGEs) are heterogeneous molecules produced by the non-enzymatic glycation of proteins, lipids, or nucleic acids during hyperglycaemia. Accumulation of AGEs in the peripheral nerves has recently been proposed as an additional risk factor for the development of diabetic neuropathy (DN). The gold standard for measurement of tissue-bound AGEs is tissue biopsy. However, their assessment with the newer, fast and simple method of skin autofluorescence (sAF) has recently gained special interest by virtue of its non-invasive, highly reproducible nature and its acceptable correlation with the reference method of skin biopsy. Accumulation of tissue AGEs evaluated by sAF has been shown to independently correlate with DN. Importantly, increasing evidence underscores their potential value as early biomarkers of the latter. Further important associations include diabetic nephropathy, diabetic retinopathy and cardiovascular autonomic neuropathy. However, the value of the implementation of screening with skin AGEs for DN remains unclear. The aim of the present review is to critically summarise current evidence on the association between skin AGEs and diabetic microvascular complications, with a particular emphasis on diabetic neuropathy, and to note the most important limitations of existing knowledge. Longer follow-up studies are also highly anticipated to clarify its role and provide data on patient selection and cost-effectiveness.

## Background

Diabetic neuropathy (DN) is a very common complication of diabetes mellitus (DM) [1, 2]. It may have several variants, but distal symmetric sensorimotor neuropathy (diabetic peripheral neuropathy, DPN) is by far the commonest form, being the most frequent neurological complication of DM [1, 2]. Moreover, we now know that it may develop early in the course of diabetes, rather than being a predominantly late complication, as previously thought [1–3]. Indeed, DN may be detectable even during the first 12 months after the diagnosis of DM, or occasionally even in prediabetes [1–3]. Despite all that, DN is still suboptimally diagnosed [1–4]. Granted that it increases morbidity and mortality, as well as being a cardinal factor in the aetiopathogenesis of the diabetic foot [1–4], improved diagnosis is a medical need.

In recent years, metabolic byproducts called advanced glycation end-products (AGEs) have attracted scientific interest [5–7]. Indeed, many studies have strongly linked them with the development and progression of DM and its complications [5, 6]. Thus, their non-invasive measurement, for instance in the skin, has been proposed in the overall diagnostic work-up of DM [5]. To this purpose, the autofluorescent properties of AGEs are employed, without discomfort for the patient [5]. Thus, an increasing number of studies has tried to establish skin AGEs as predictors of microvascular diabetic complications. Nonetheless, to the best of our knowledge, a systematic estimation of this relationship has not been performed during the last decade, and associations remain rather controversial [8].

In this context, the purpose of the present brief narrative review is to critically summarise current knowledge on the relationship between skin AGEs and diabetic neuropathy with a brief reference to the other microvascular complications of DM. Indeed, some recent publications during the last 3 years have provided interesting additional evidence in this field, allowing us to re-examine the subject.

## Advanced glycation end-products

AGEs represent a rather heterogeneous class of molecules produced by the non-enzymatic glycation of proteins, lipids, or nucleic acids during a hyperglycaemic state [5, 6]. The reaction resulting in the formation of AGEs is known as the “Maillard reaction” [5]. AGEs then undergo extracellular proteolysis followed up by intracellular uptake by tissue macrophages, and are finally excreted by the kidneys [5, 6].

AGEs can accumulate with age. However, their accumulation is exacerbated by some pathologic conditions such as DM, cardiovascular disease, Alzheimer’s disease etc. [5–7]. Food products which undergo heat-processing are also a significant source of AGEs: the latter are partially absorbed during ingestion [6, 7]. These food products also produce Maillard reaction products, including AGEs [6, 7]. Reduction in cooking temperature has been shown to decrease AGE levels, as measured by calculating levels of carboxymethyl-lysine (CML) [6]. Moreover, pH levels play a major role in the development of AGEs and pretreating food products with acidic substances such as vinegar and lemon have been shown to reduce AGEs [6, 7].

AGEs can be absorbed by simple diffusion if found in their free form. However, the absorption of their protein-bound form is less successful [7]. As confirmed by measurement of faecal AGEs, individuals who have had pentosidine in its free form (in coffee) absorb more AGEs when compared with those ingesting it in its protein-bound form (in bread) [7].

Interestingly, coffee consumption has also been shown to have a significant and dose-dependent correlation with sAF levels in subjects without DM [9]. However, in individuals with type 2 DM (T2DM), there was a non-significant trend [9]. Other than dietary sources, smoking has also been found to be a major source of exogenous AGEs [10]. In individuals who were past or current smokers, sAF levels were higher compared with never smokers, and this did not depend on the presence or not of DM [10]. As was the case with coffee consumption, sAF levels were closely associated with number of packs/years, which strongly suggested a dose-dependent effect [10]. These results have been confirmed in another study, in which it was additionally shown that smoking cessation results in a gradual decrease of sAF levels [11].

## DN

Traditionally, DN is classified into diffuse and focal [1, 4]. It may affect the somatic or the autonomic part of the peripheral nervous system [1, 4]. In the latter case, it is called diabetic autonomic neuropathy (DAN), and its most important manifestations are seen in the gastroinstestinal and the cardiovascular system [1, 4, 12]. Overall, the commonest form of DN is, as aforesaid, DPN [1, 4].

**Fig. 1:**
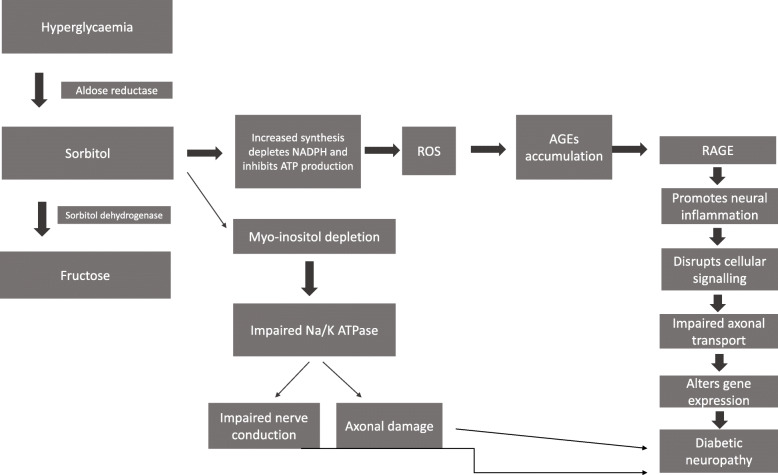
The contribution of AGEs to the pathogenesis of diabetic neuropathy. A major mechanism includes activation of the polyol pathway, leading to NADPH depletion and increased production of reactive oxygen species (ROS). The latter contribute to AGEs accumulation. AGEs act via the receptor of advanced glycation end products (RAGE), promote neural inflammation, impair neuronal electrical activity and induce unfavourable gene changes. These perturbations, along with direct neuronal toxicity from intracellular sorbitol accumulation, culminate in diabetic neuropathy. AGEs: advanced glycation end products; NADPH: Nicotinamide adenine dinucleotide phosphate; RAGE: receptor of advanced glycation end products; ROS: reactive oxygen species

The main risk factors of DN include DM duration, marked hyperglycaemia, dyslipidaemia, hypertension, smoking and age [13]. Its pathogenesis is hugely complex and can be seen as an incompletely understood conundrum of hyperglycaemia, insulin resistance, chronic inflammation, oxidative stress, dyslipidaemia, endothelial dysfunction and (in the case of type 1 DM) inadequacy of insulin [1, 4]. Nowadays, an additional causative factor is considered to be the accumulation of AGEs in the peripheral nerves, increasing reactive oxygen species (ROS) and, finally, promoting neural inflammation and impairing axonal transport (Fig. 1). Such accumulation has been associated with lower limb sensory loss [14].

DPN typically presents as chronic insidious progressive symmetrical sensory loss in the distal parts of the lower extremities (so called “stocking and glove distribution”) [1, 3, 4]. This relates to temperature, pain, light touch and vibration perception [1, 4]. In more advanced cases, motor deficits (muscle weakness, attenuation of Achilles reflexes) manifest as well. Moreover, impaired sweat gland innervation renders the skin dry and fragile [1, 4]. Most patients are asymptomatic, while the combination of these deficits places the feet at increased risk of foot ulceration through unrecognised trauma [1, 3, 4]. Occasionally, patients may complain of numbness pain or discomfort, which may exhibit nocturnal exacerbation [1, 4].

The diagnosis of DN rests on clinical examination, depending on the system affected [1, 4, 15]. For example, cardiovascular reflex tests are used to diagnose cardiovascular autonomic neuropathy [1, 16]. For DPN, simple bedside clinical examination assessing sensory and motor nerve fibres is used [1, 15]. More sophisticated methods are mainly used for difficult cases, differential diagnosis or research purposes [1, 15]. To enable wider DPN screening, some simple new diagnostic tools have been developed as well: the most important of these are automated sural nerve conduction study [17, 18] and the indicator test Neuropad assessing skin dryness by means of a very simple colour change [18].

The therapeutic principles of DN may be summarised as follows: optimisation of glycaemic control, treatment of other vascular risk factors (dyslipidaemia, hypertension, smoking), pathogenesis-oriented treatment, analgesia as required and avoidance of complications (such as foot ulceration) [1, 4, 19]. There has been considerable progress in pain relief [1, 4, 19]. Conversely, pathogenesis-oriented treatment is still suboptimal and only alpha-lipoic acid has shown efficacy [4, 19].

## Measurement of skin AGEs

AGEs are currently measured by means of Enzyme-linked immunosorbent assay (ELISA) using monoclonal or polyclonal antibodies, high performance liquid chromatography (HPLC) and mass spectrography [5, 9]. However, the gold standard for measurement of tissue-bound AGEs is undoubtedly tissue biopsy. This method is highly accurate and provides great insight into disease process, but it is invasive and takes a much longer period compared to the newer methods [5, 9].

**Fig. 2:**
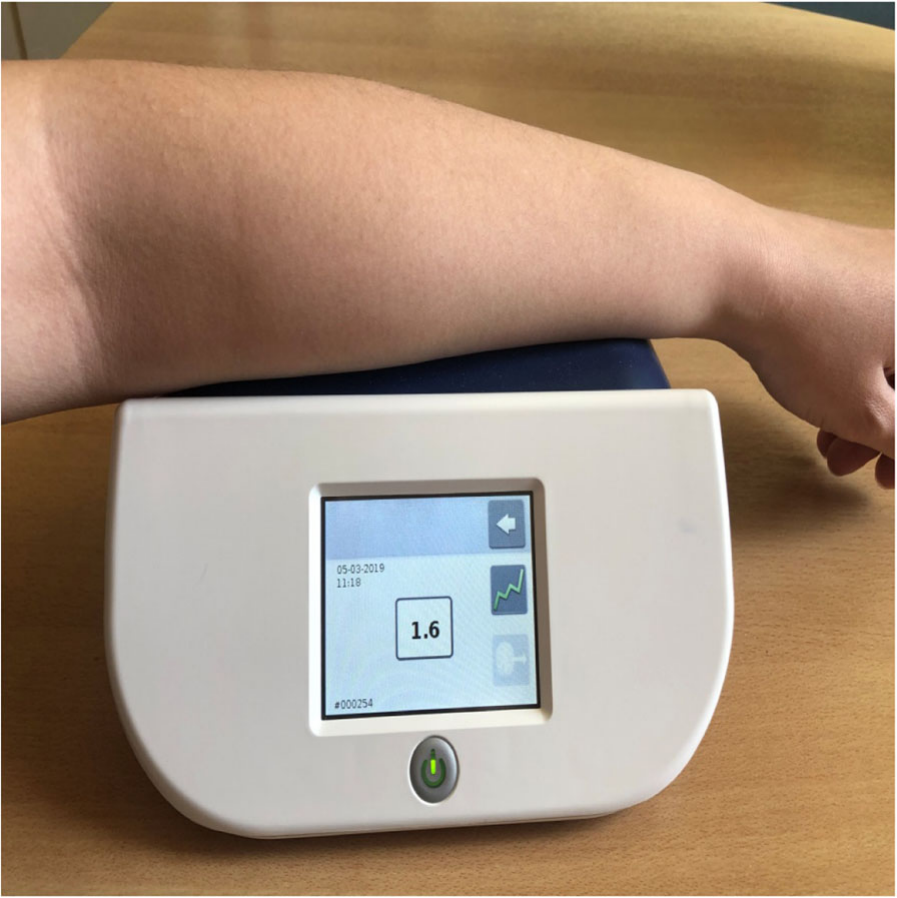
Skin autofluorescence, as measured by the autofluorescence reader AGE reader mu connect (Diagnoptics, NL). Local creams should not have been applied for 12 h. The examinee places the forearm of his/her dominant upper limb on the silicone armrest of the device. In this armrest, there is a small examination window for selection of a skin area. The elbow should be aligned with the edge of the armrest and no movements should be made during measurement. The examiner switches on the device using the power button. Activation is performed through the menu on the touch screen. AGEs result is shown on the screen. Approximate measurement duration is 30 s. Interpretation of results may be facilitated by available age- and sex-adjusted normative data

Skin autofluorescence is measured using an autofluorescence reader (e.g. AGE reader mu connect; Diagnoptics, NL) (Fig. 2), which illuminates a small portion of the skin (approximately 4 cm^2^) on the volar side of the examinees’ forearm [5, 20]. The device produces light on the selected area with an excitation light source of ∼370 nm. The emission light and reflected excitation light emanating from the skin is then measured using a glass fibre in the 300-600 nm range [20]. These values are then computerised and through a series of calculations produce values in arbitrary units (AU). Meerwalt et al. [21] have shown that measurements by the AGE reader exhibited adequate correlations with readings from skin biopsies for specific AGEs, such as pentosidine, CML and carboxyethyl lysine (CEL) [5].

## Skin AGEs and diabetic microvascular complications

In recent years, skin AGEs have been utilised more frequently for diagnostic purposes as they offer a non-invasive, and inexpensive diagnostic tool with a high degree of reproducibility. Increased skin AGEs has been thoroughly studied in the context of microvascular complications of diabetes mellitus (Table 1) [5, 22–33].

**Table 1:** Human studies investigating the association between skin AGEs and microvascular complications other that diabetic neuropathy

First Author (year)	Participants and diabetes type	Aggregate clinical outcome	Main findings
**Retinopathy**			
Gerrits (2008) [22]	973 T2DM	Retinal photography	Multivariate analysis (adjustment for sex, diabetes duration, HbA_1c_, current smoking, systolic blood pressure, HDL cholesterol, LDL cholesterol, triglycerides, BMI): no association
Chabroux (2010) [23]	133 T1DM	Retinal photography	Multivariate analysis (adjustment for age, diabetes duration, HbA_1c_, smoking, retinopathy, nephropathy and neuropathy): no association
Sugisawa (2013) [24]	210 T1DM/110 controls	Retinal or fundus photography	Multivariate analysis (adjustment for age at registration, age at onset of diabetes, duration of diabetes, sex, BMI, and sAF): positive association with retinopathy; positive association with retinopathy severity
Yoshioka (2018) [25]	162 T2DM/42 controls	Diagnosed by independent ophthalmologists according to a position statement by the American Diabetes Association (no further information in the text)	Multivariate analysis (adjustment for age, diabetes duration, HbA_1c_, serum pentosidine concentration and eGFR): positive association; positive association with retinopathy severity
Bentata (2017) [26]	444 T2DM (17% with chronic renal insufficiency)	Fundus examination or retinography or (if required) optical coherence tomography	Multivariate analysis (adjustment for age, duration of diabetes, hypertension, insulin, HbA_1c_ and eGFR): no association of sAF and retinopathy among subjects with T2DM and renal insufficiency; positive association among subjects with T2DM and eGFR≥60 ml/min/m^2^
Tanaka (2012) [27]	130 T2DM	Retinal photography	Positive association with proliferative diabetic retinopathy but not with simple diabetic retinopathy
Yasuda (2015) [28]	67 T2DM/67 controls	Fundus photography	Association with presence and severity of retinopathy
Hirano (2014) [29]	138 T2DM/111 controls	Ophthalmoscopy and contact lens slit lamp biomicroscopy	Multivariate analysis (adjustment for sex, age, HbA_1c_, self-stated duration of diabetes, systolic and diastolic blood pressure, history of smoking, diabetic nephropathy, and diabetic neuropathy): association with retinopathy severity; no association with diabetic macular oedema
Noordzij (2012) [30]	563 T2DM	Retinal photography or fundoscopy	Multiple linear regression: no association
**Nephropathy**			
Gerrits (2008) [22]	973 T2DM	Diabetes-associated (micro) albuminuria defined as an ACR > 2.5 mg/mmol for men and > 3.5 mg/mmol for women	Multivariate analysis (adjustment for sex, diabetes duration, HbA_1c_, current smoking, systolic blood pressure, HDL cholesterol, LDL cholesterol, triglycerides, BMI): positive association
Chabroux (2010) [23]	133 T1DM	Detection of (micro)albuminuria	Multivariate analysis (adjustment for age, diabetes duration, HbA_1c_, smoking, retinopathy, nephropathy and neuropathy): positive association
Sugisawa (2013) [24]	210 T1DM/110 controls	First method: chronic kidney disease stages 1–5 defined by National Kidney Foundation practice guidelines; second method: *1*) normoalbuminuria (ACR < 30 mg/g Cr); *2*) incipient nephropathy (ACR ≥30 but < 300 mg/g Cr); *3*) overt nephropathy (ACR ≥300 mg/g Cr); *4*) chronic renal failure (serum Cr > 2.0 mg/dL); and *5*) renal failure on dialysis	Multivariate analysis (adjustment for age at registration, age at onset of diabetes, duration of diabetes, sex, BMI, and sAF): positive association with nephropathy; positive association with severity of nephropathy
Yoshioka (2018) [25]	162 T2DM/42 controls	Classified as: (1) pre-nephropathy (stage 1) (ACR < 30 mg/g Cr); (2) incipient nephropathy (stage 2) (30 ≤ ACR < 300 mg/g Cr); (3) overt nephropathy (stage 3) (ACR ≥ 300 mg/g Cr); (4) kidney failure (stage 4) eGFR < 30 ml/min/1.73^2^; and (5) dialysis therapy (stage 5)	Multivariate analysis (adjustment for age, diabetes duration, HbA_1c_, serum pentosidine concentration and eGFR): no association; positive association with incipient nephropathy (stage 2) but not with overt nephropathy (stage 3)
Rajaobelina (2017) [31]	154 T1DM	Chronic kidney disease (eGFR < 90 ml/min) and/or albuminuria	Increase of sAF (by at least 10%): linked with deterioration of microalbuminuria and of eGFR
Tanaka (2012) [27]	130 T2DM	Positive dipstick results for proteinuria (≥1 +)	Positive association with proteinuria ≥3.5 g/gCr
Noordzij (2012) [30]	563 T2DM	ACR > 3.5 in women and > 2.5 in men	Multivariate analysis: no association

*ACR* Albumin-to-creatinine ratio, *BMI* body mass index, *Cr* creatinine, *eGFR* estimated glomerular filtration rate, *HbA*_*1c*_ glycated haemoglobin, *HDL* high density lipoprotein, *LDL* low density lipoprotein, *SAF* skin autofluorescence, *T1DM* type 1 diabetes mellitus, *T2DM* type 2 diabetes mellitus

In the case of retinopathy, apoptosis of retinal capillary pericytes and endothelial cells (a major contributor to the pathogenesis of this complication) has been linked with AGEs [5]. In alloxan-induced diabetes, AGE-specific modifications of the basement membrane proteins of retinal capillaries were observed [32]. Another group reported that increased AGEs are linked with vascular endothelial growth factor (VEGF) overproduction (another major pathogenetic mechanism) [32]. Nevertheless, previous evidence is rather controversial, with a number of studies reporting lack of association with skin AGEs [23, 30, 34], others reporting a clear independent correlation with retinopathy [24, 25, 28] and disease severity [29], whereas, interestingly, macular oedema seems to develop independently of any increase in the levels of skin AGEs (Table 1) [26, 29].

In diabetic nephropathy, AGEs can affect extracellular matrix (ECM) protein in the glomerular mesangium and tubulointerstitium [5, 35]. This is mediated by alterations in protein structure and composition, as well as increase in matrix formation [5, 14]. The latter is attributable to the fact that proteins become more resistant to enzymatic digestion. A potential role of AGEs in the development of diabetic nephropathy by influencing the renin-angiotensin system has also been suggested. This interaction has been demonstrated in vitro by the reversal of AGE-induced collagen formation with captopril [31]. Nonetheless, previous studies do not unanimously support the correlation between nephropathy and skin AGEs (Table 1) [22–25, 27, 30, 31].

## Skin AGEs and DN

Accumulation of tissue AGEs evaluated by sAF was independently associated with DPN (diagnosed by the presence of at least two of the following: sensory symptoms, bilaterally decreased or absent ankle reflexes, and decreased vibration perception in bilateral medial malleoli) among 193 T2DM persons following correction for known risk factors of microvascular complications [36]. Wan et al. [37] have further demonstrated that increased sAF levels are directly correlated with impairments in nerve conduction velocity and nerve conduction amplitude. In T2DM individuals with sAF levels above 2.2 AU, there was a significant decrease in sensory nerve conduction velocity and compound action potential of the sural and superficial peroneal neve [37]. Importantly, sAF greater than 2.7 AU exhibited a further significant negative correlation with deterioration of the aforementioned parameters, as compared with sAF levels ≤ 2.2 AU and sAF 2.2–2.7 AU [37]. Additionally, in a multi-centre study including 497 T1DM and T2DM humans, sAF levels significantly increased with the clinical severity of DPN, as graded by the Toronto Clinical Neuropathy Score [38].

A further prospective 4-year study confirmed the value of sAF to predict the development of DPN in a middle-aged T1DM cohort with long-standing T1DM [31]. Even after adjustment for multiple confounding factors (age, sex, height, body mass index, tobacco, glycated haemoglobin, diabetes duration, estimated glomerular filtration rate and albumin excretion rate), each standard deviation change in baseline sAF was correlated with an almost 3-fold increased risk of developing signs of DPN [31]. The latter were defined by the occurrence of neuropathic pain and/or feet sensory loss, foot ulceration or neuropathic osteoarthropathy 4 years later [31]. As regards neuropathic symptoms, the potential contribution of AGEs to the development of painful DPN, may be partly explained on the basis of previously published evidence suggesting that AGEs (mainly methylglyoxal) may cause diabetic neuropathic hyperalgesia by increasing the electrical excitability and facilitating firing of nociceptive sensory neurons [39].

Impressively, there is also evidence of increased sAF levels before the onset of DPN symptoms. This further reinforces the notion that altered sAF could precede the progression of DPN. Indeed, a study has shown that sAF was correlated negatively with nerve conduction velocity and nerve conduction amplitude of the median, peroneal and sural nerve in humans with T1DM or T2DM even without the clinical manifestations of DPN [38]. Here DPN was defined using the Dutch Diabetic Neuropathy Symptoms scale, the Dutch Diabetic Neuropathy Examination scale, and quantitative sensory function testing with Semmes-Weinstein monofilaments (SWMF) [34]. These results were replicated in a longitudinal study involving 881 well-controlled T2DM participants [22]. After a mean follow-up of approximately 3 years, the relationship between sAF and development of DPN (diagnosed by SWMF) remained significant after adjustment for T2DM duration [22].

Furthermore, high sAF levels have been observed in individuals with diabetic foot ulcers [40, 41]. However, the highest sAF quartile has been independently associated with increased VPT levels even before reaching a VPT indicating high risk of foot ulceration [40, 41]. In the same context, an inverse relationship between high sAF levels and lower electrochemical skin conductance has been demonstrated, even in the normal range of sudomotor function, thereby providing evidence that increased sAF levels may even precede small-fibre sudomotor dysfunction [31].

Finally, Orchard et al. [35] have shown a strong association between sAF and CAN, as assessed by sinus arrhythmia. This extends the importance of AGEs from DPN to CΑΝ as well, although the literature is rather controversial (Table 2) [34–36, 42].

**Table 2:** Human studies investigating the association between skin AGEs and cardiovascular autonomic neuropathy

First Author (year)	Participants and diabetes type	Method of assessment of CAN	Main findings
Osawa (2018) [36]	193 T2DM/24 controls	Heart rate variability	Multivariate analysis (adjustment for age, sex, HbA_1c_, T2DM duration, BMI, hypertension, dyslipidaemia and smoking history): positive association
Meerwaldt (2005) [34]	33 T2DM and 13 T1DM/21 controls	Heart rate variability	Positive association
Orchard (2013) [35]	1185 T1DM	Heart rate variability	Positive association before adjustment for mean HbA_1c_ over time; insignificant association after adjustment
Conway (2011) [42]	111 T1DM	Electrocardiographic abnormal heart rate response to deep breathing	Multivariate analysis (adjustment for age and updated mean, 18-year average, HbA_1c_): positive association

*BMI* body mass index, *HbA*_*1c*_ glycated haemoglobin, *T1DM* type 1 diabetes mellitus, *T2DM* type 2 diabetes mellitus

## Clinical implications

Measurement of skin AGEs has become very easy and reproducible with modern technology. Indeed, previous methods of AGEs measurement, although accurate and useful for diagnostic purposes, were invasive, expensive and therefore unsuitable for widespread use. The availability of skin AGEs has enabled their consideration as markers of microvascular diabetic complications. Indeed, should increased skin AGEs be demonstrated unequivocally to herald the development and/or deterioration of these complications, they might prove useful in their early detection. Thus, they might be expected to contribute to closer follow-up of high-risk individuals, early treatment initiation and avoidance of further deterioration, perhaps also improved quality of life [43].

So far, AGEs have been shown to correlate with DPN, CAN, diabetic retinopathy and nephropathy. In DN, they are being increasingly appreciated as promising early markers of this complication. They appear to become increased before the impairment of nerve conduction parameters, onset of symptoms or increase in vibration perception threshold. This evidence notwithstanding, important limitations of the literature pertain to differences in the populations studied and various diagnostic criteria of DPN (Table 3).

**Table 3:** Key findings of the studies assessing the association between skin AGEs and DPN

1. Accumulation of skin AGEs is unanimously associated with DPN [36–38] and with diabetic foot ulceration [40, 41]	
2. Increasing sAF levels predict the development of impendent DPN [22, 31, 38]	
3. Evidence supports that the increase in skin AGEs may precede small-fibre sudomotor dysfunction [31] and altered vibration perception threshold [40, 41]	
4. Important limitations of published studies include: populations with different diabetes types; application of varying definitions for DPN diagnosis; divergent implementation of sAF cut-off values	
5. Future studies are eagerly anticipated to clarify important issues, namely improvement in DN as a result of AGEs screening and the cost-effectiveness of the latter	

*AGEs* advanced glycation end products, *DPN* diabetic peripheral neuropathy, *sAF* skin autofluorescence

The question, then, arises as to whom, when and how often we shall screen. Ideally humans with DM should be offered early measurement of skin AGEs as screening of DN [44]. However, despite the easy-to-use devices, it is not possible to screen all DM individuals, and so, for the time being, it may be enough to increase AGEs measurements. Moreover, one might argue that while skin AGEs are correlated with incipient nerve fibre impairment, the improvement in DN as a result of AGEs screening remains a speculation and has not yet been demonstrated. Indeed, we would like to know more on this issue, including the cost-effectiveness of widespread screening [45]. Finally, longer follow-up of patients with early increases in skin AGEs is highly welcome. Clarification of all these issues will help our decisions on the implementation of AGEs measurement in everyday reality, with the aim to improve outcomes of DPN, a still valid strategic priority [46–49].

One may be tempted to even extend these thoughts to the complications of DN, primarily diabetic foot ulcers (DFUs) [1, 2, 48]. Treatment of the latter is still based on revascularisation (if needed), debridement, infection control and off-loading, while other measures are used secondarily [50]. This therapeutic approach has not changed, because no other intervention has demonstrated superiority [50]. There is also some progress in utilisation of screening tools to predict future development of DFUs, and, very recently, the indicator test for sudomotor function Neuropad has emerged as useful in this prediction [51]. One step earlier, timely identification of subjects at high risk of developing DN would also contribute to the identification of subjects at high risk of DFUs [50, 51]. Accordingly, skin AGEs measurement might prove useful as a surrogate marker of DFUs risk and, thus, contribute to meticulous treatment and education of subjects with this risk. This possibility notwithstanding, the potential utility of skin AGEs needs to be shown in practice.

## Conclusions

Skin autofluorescence represents a non-invasive, inexpensive, highly reproducible method of measuring AGEs. Increased skin AGEs have been linked with microvascular complications of DM. In retinopathy, some correlation has been shown between AGEs and progression of this complication. Additionally, skin AGEs are being increasingly appreciated as predictors of nephropathy and DN. They appear to become increased before the impairment of nerve conduction parameters, onset of symptoms or increase in vibration perception threshold.

## Data Availability

Not applicable, because this is a narrative review without original research data.

## Abbreviations

ACR: Albumin-to-creatinine ratio
AGEs: Advanced glycation endproducts
BMI: Body mass index
CML: Carboxymethyl-lysine
Cr: Creatinine
DM: Diabetes mellitus
DN: Diabetic neuropathy
DPN: Diabetic peripheral neuropathy
eGFR: estimated glomerular filtration rate
ELISA: Enzyme-linked immunosorbent assay
HbA_1c_: Glycated haemoglobin
HDL: High density lipoprotein
HPLC: High-performance liquid chromatography
LDL: Low density lipoprotein
NADPH: Nicotinamide adenine dinucleotide phosphate
RAGE: Receptor of advanced glycation end products
ROS: Reactive oxygen species
SAF: Skin autofluorescence
T1DM: Type 1 diabetes mellitus
T2DM: Type 2 diabetes mellitus
